# Unraveling Evolutionary Insights into AVT Peptide Conservation and Antimicrobial Motif Prediction Across Taxa

**DOI:** 10.3390/ijms26168026

**Published:** 2025-08-19

**Authors:** Ganesan Nagarajan

**Affiliations:** 1 Department of Biological Sciences, College of Science, King Faisal University, Al-Ahsa 31982, Saudi Arabia; nadimoolam@kfu.edu.sa; 2 Department of Basic Sciences, Preparatory Year, King Faisal University, Al-Ahsa 31982, Saudi Arabia

**Keywords:** arginine vasotocin (AVT), epinephelus coioides, neuroendocrine, gene ontology, antimicrobial peptide, cationic peptide

## Abstract

Arginine vasotocin (AVT) is well known for its role in steroidogenesis and estradiol biosynthesis during early brain development in *Epinephelus coioides*. Despite its hormonal functions, the biological significance of AVT across different taxa remains poorly understood. Hence, the present study aims to unravel the evolutionary conservation and functional annotation of AVT in different taxa. Additionally, the antimicrobial properties of AVT were investigated across multiple conserved domains. From the sequence comparison results, AVT is highly conserved and a core motif across teleosts, mammals, plants, and bacteria, suggesting functional constraints under strong evolutionary selective pressure. Phylogenetic analyses highlighted AVT and its homologs evolved from a common ancestral gene. The functional enrichment analyses of the genes revealed different taxa that share an analogy with AVT genes. The major pathways for AVT and its homologs are identified in neuroendocrine, immune, and stress signaling. Importantly, a conserved AMP-like motif within the AVT sequence (GIRQCMSCGPGDRGR) was identified. The motif is predicted for its potential role in membrane permeabilization and antimicrobial defense. Physicochemical properties of this peptide showed cationic and amphipathic features, with cysteine residues conferring structural stability. Overall, the results underscore the pleiotropic role of AVT across different taxa, showing its evolutionary stability. AMP-like AVT motif was predicted as a promising candidate for synthetic peptide design. Experimental evaluation with peptides will determine their antimicrobial potential in infection models.

## 1. Introduction

Arginine vasotocin (AVT) hormone is found in different non-mammalian species such as fishes, amphibians, reptiles, and birds [1,2]. The mammalian hormone equivalent to AVT is the arginine vasopressin (AVP) [3]. Both AVT/AVP hormones are produced in the brain and belong to the neuropeptide family [4]. The presence of AVT in aquatic organisms such as fish is essential for maintaining the level of salt and water in their bodies [5,6]. AVT has been studied for its potential implications in developing dominance and aggressiveness across the vertebrate taxa [7]. In teleost fish, the dominant–subordinate status was established by preoptic area neurons [8]. An increase in both parvocellular and magnocellular AVT neurons was essential for establishing the aggression in dominant fish [7,9]. The other AVT-related peptide, called isotocin, similar to mammalian oxytocin, is essential to control blood pressure and acts as an antidiuretic hormone [10]. AVT and AVP hormones bind to specific receptors to initiate various functions such as social behavior, stress response [11,12], osmoregulation [13], and reproduction [14]. In teleost fish, AVTR1 receptors are essential for binding AVT and are found not only in the brain but also in tissues like the gills, kidneys, gonads, and digestive system [13,15]. The receptor V1a-type vasopressin and oxytocin/mesotocin are expressed mainly in the brain of mammals. In fish, amphibians, and birds, the receptor V1a-type vasopressin is expressed in the central nervous system. In birds and mammals, the expression of the vasopressin receptor influences the difference in social behavior [16]. Genome mutations have directed isoforms of the receptors in Actinopterygians, finned ray fishes. The V1a vasotocin receptor has two isoforms (v1a1 and v1a2) and two V2-type receptors (v2a and v2b). In Cyprinodon nevadensis amargosae, the expression levels of four vasotocin receptors, the isotocin receptor (itr) and pro-vasotocin and proisotocin are responsible for the aggressive behavior in the social classes of the organism [10]. The anatomical features of AVT/AVP and their role in behavioral and physiological functions are well-known in individual species [14,17,18]. However, their ubiquitous species diversity has not been explored, making them the subject of studies spanning different taxa.

In the present study, AVT from *E. coioides*, a range-spotted grouper, was used as the target for investigation. *E. coioides* is a marine fish cultured due to its high market value. *E. coioides* is an ideal model for studying sex differentiation and reproduction [19]. *E. coioides* has been investigated for its role in steroidogenesis [20], particularly in enhancing estradiol biosynthesis essential for early brain development [21]. Moreover, an investigation with exogenous AVT has induced the expression of steroidogenic enzymes and estrogen receptors both at the cellular and gene level, revealing their regulatory role in estradiol biosynthesis and early brain development [9]. The role of AVT is well-known in the neurohormonal network orchestrating reproduction and neural development [9]. However, the role of AVT for its potential in immune responses and host defense remains underexplored [22]. This study aims to investigate the evolutionary conservation, functional annotation, and antimicrobial potential of AVT across diverse species. Through phylogenetic analyses, the evolutionary pressure on AVT among different species of vertebrates, invertebrates, plants, and bacteria will be examined. By integrating phylogenetic, motif, and pathway analyses, the novel insights into AVT’s possible role as an antimicrobial peptide and its association in the neo-functionalized pathways will be uncovered.

## 2. Results

### 2.1. Sequence Retrieval

AVT is a neuropeptide from non-mammalian vertebrates with various physiological and behavioral processes. The protein sequence of AVT from *E. coioides* was retrieved from NCBI GenBank with an accession ID ADF36550.1. The sequence length of 153aa features signal peptide coding (1 to 19aa), arginine vasotocin product (20 to 29aa), and a hormone-associated region (39 to 117) corresponding to the C-terminal domain of neurohypophysial hormone. The retrieved sequence from the GenBank database was subjected to various functional analyses.

### 2.2. Prediction and Analyses of Antimicrobial Peptide (AMP)

The screened peptide sequence of AVT revealed 41 regions corresponding to an antimicrobial peptide. Based on the predicted peptide score, nine peptides were obtained with a score > 1. The peptide sequence of GIRQCMSCGPGDRGR at the start position of 37 was the top hit, with a score value of 2.146 predicted as the top hit. Physicochemical properties of the peptide sequence were observed with a molecular weight of 1592.83 Da. The theoretical isoelectric point (pI) was observed as 9.02. One negatively charged residue (Asp) and three positively charged residues (Arg) were observed in the peptide sequence. The aliphatic index was observed as 26.00, indicating the lower thermostability of the peptide. The GRAVY score of −0.873 represents the hydrophilic nature of the peptide. The distribution of amino acids, such as 10 polar residues and 5 non-polar residues, indicates the peptide is highly water soluble. GLN, SER, and GLY in the peptide sequence can contribute to the flexibility and hydrogen bonding potential. The presence of the positive residues (Arg and Asp) in antimicrobial peptides enhances electrostatic interactions with negatively charged bacterial membranes, determining the charge for the peptide (net charge +2) (Table 1).

**Figure 1:**
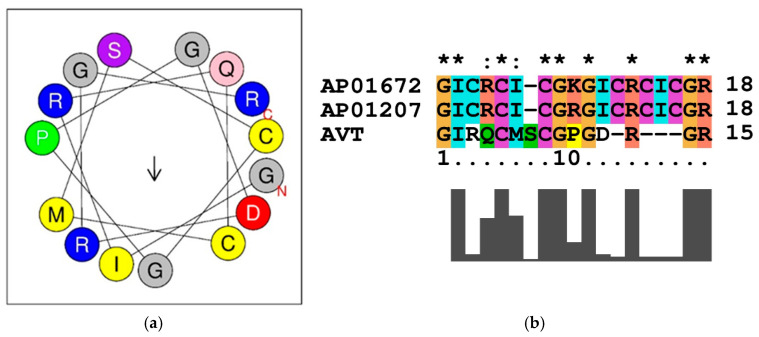
(**a**) The helical wheel projection of a peptide sequence show the distribution of amino acid residues around an alpha-helix. The amino acids are arranged in a circle, each representing a turn in the helix (per turn 3.6 residues). The arrow indicates the direction of the helix axis. The blue color (R) represents the positively charged (arginine) residue. The red color (D) indicates the negatively charged aspartic acid residue. The yellow (C, M, I) color indicates the hydrophobic residues (cysteine, methionine, isoleucine). The green (P) color represents the proline (structurally unique, often induces kinks) residue. The grey (G, G, G) color code represents the glycine (small, flexible) residue. The pink (Q) color represents the polar, uncharged (glutamine) residues and purple (S) color represents the polar, uncharged (serine) residues. The C and N represent the C-terminal and N-terminal regions of the peptide. (**b**) Sequence similarity of AVT-peptide with RC-101 (AP01672) and Retrocyclin-1 (AP01207) represents the hydrophobic (G, I, and C) and cationic residues (R), which are conserved in the functional domain of the peptides and are crucial for antimicrobial activity. “*”/“**” indicates the identity of the aminoacid in the sequence alignment. “:” indicates the high similarity with substitution of aminoacids in the position of the sequence alignment.

The mean hydrophobicity (0.185) and hydrophobic moment (0.210) values obtained through the HeliQuest server indicate that the peptide sequence is hydrophilic and mildly amphipathic. Additionally, the helical wheel prediction for the peptide sequence revealed the peptide with an alpha helix (Figure 1a). The wheel shows that the presence of two CYS residues is responsible for the disulfide bond formation and contributes to structural stability. Similarly, the PRO residue can induce structural bends or kinks in peptide chains.

The predicted peptide sequence was searched for sequence similarity in APD3 database. AVT peptide shares 50% sequence similarity with RC-101 and Retrocyclin-1. The peptide RC-101 is a derivative of Retrocyclin-1, which particularly shares a high cysteine content and beta-sheet structure required for stabilizing the disulfide bridges. The sequence similarity analyses of AVT shows high similarity with the region of cationic and hydrophobic residues (Figure 1b) crucial for antimicrobial function. Notably, previous in vitro investigations with RC-101 and Retrocyclin-1 have been shown to exhibit broad-spectrum antimicrobial activities against Gram-positive bacteria (*Staphylococcus aureus* USA300), Gram-negative bacteria, viruses (HIV-1, SARS-CoV-2, Influenza), and biofilms [23,24,25].

### 2.3. Motif Prediction in AMP Sequence

**Figure 2:**
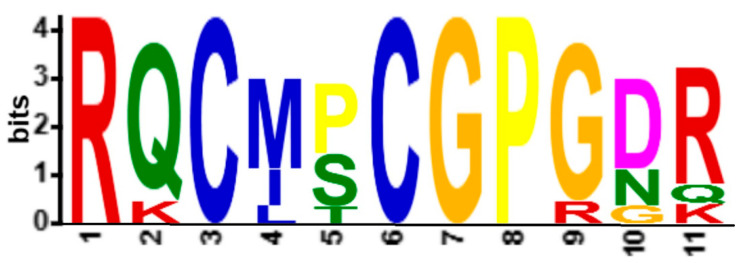
The consensus motif identified in the AVT peptide sequence. Using MEME, the consensus motif RQCMSCGPGDR was identified in 36 protein targets with a *p* value of 2.75 × 10^−65^.

Motifs are an approximate sequence pattern that occurs repeatedly in a group of related sequences and provides structural and functional information about the protein sequence. Prediction of motifs from AVT sequences enables functional annotation across different genera. From MEME, the consensus sequence among the multiple sequence alignments was analyzed and revealed the motif sequence RQCMSCGPGDR (RQCMxCGPGxGR), with a length of 11 amino acids. Pattern analyses of the motif sequence shows that highly conserved arginine (R) indicates strong functional or structural importance. The presence of the cysteine-glycine-proline-glycine segment reveals the loop or hairpin-like structures, which may be involved in protein-protein interaction. The presence of aspartic acid (D) and arginine (R) in the C-terminal region forms a charged patch with potential active binding site functionality (Figure 2). The low *p*-values detected across multiple sequence entries suggest the motif pattern may be a functionally relevant motif. Based on the annotation, the motif sequence might have a specific role in protein interaction or signaling.

### 2.4. Sequence Similarity Analyses

The identified peptide sequence was searched for its similarity with other organism databases. The protein sequence showing similarity to the AVT peptide sequence of *E. coioides* were subjected to multiple sequence alignment. The query peptide has significant sequence conservation with a broad range of organisms spanning from bacteria to vertebrates (Table 2). The alignment resulted in 100% peptide sequence identity with six different genera of organisms, *Catostomus commersonii*, *Arabidopsis thaliana*, *Gloeobacter violaceus*, *Drosophila melanogaster*, *Psychrobacter arcticus*, and *P. cryohalolentis*. Among vertebrates, *Takifugu rubripes* (93.33%), *Oncorhynchus masou* (92.31%), and *Danio rerio* (87.50%) have shown sequence identity. The vasotocin-neurophysin and isotocin-neurophysin precursor sequences were closely related to the fish species. The amino acid pattern conservation reveals that peptide has a significant role in osmoregulation and social behavior across aquatic species. The conservation also extended to other vertebrates like mammals. The lower identity (~64–85%) among the mammals reflects moderate evolutionary divergence by retaining core structural features. Arginine decarboxylase protein from *Bacillus cereus* and *B. anthracis* showed ~77.78% identity, indicating conserved domains that may regulate basic cellular functions. Species-specific divergence was observed among amphibians, birds, and mammals, with the identities ranging from 66.67% to 64.29%, which may reflect functional specialization. The results for sequence alignment suggest a highly conserved peptide motif that may have functional and structural significance across different taxa. Both aquatic and terrestrial organisms showed significant sequence conservation, indicating the evolutionary stability of the AVT peptide sequence.

### 2.5. Phylogenetic Analyses

**Figure 3:**
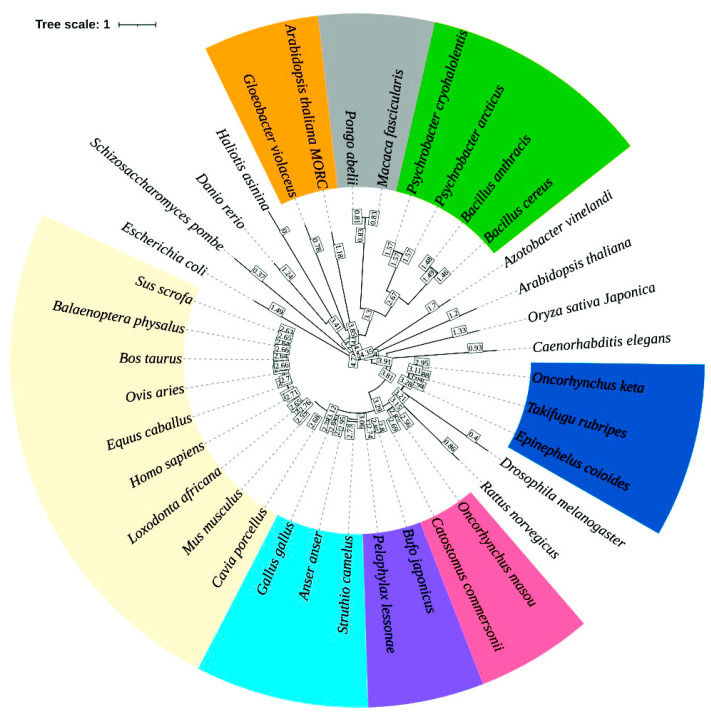
Phylogenetic analyses of AVT peptide sequences across diverse taxa (teleost fishes, amphibians, reptiles, birds, mammals, invertebrates, plants, fungi, and bacteria) was performed using the ML method. The tree was constructed using the JTT model, with 1000 bootstrap replicates. The bootstrap values are represented in a box on each node. The circular rooted tree (Tree scale: 1) represents the branch lengths, signifying the evolutionary divergence and conservation of AVT and AVT-related sequences across kingdoms. The smallest branch distances are observed among closely related species (e.g., mammals, teleosts) and larger branch distances among bacteria and plants. Different color-coded clades represent the taxonomic groups used in the analyses.

A phylogenetic tree was constructed using a multiple sequence alignment of the AVT peptide sequence and its homologous sequences from diverse genera. The AVT phylogenetic tree was annotated for the different clade and their closeness using the iTOL web server (version 7). The circular tree illustrated the evolutionary relationships among diverse taxa, including bacteria, invertebrates, plants, and vertebrates. In the evolutionary tree, branch length determines the degree of divergence and bootstrap values represent the reliability of the inferred clades. The tree topology reveals distinct clustering patterns, in which *E. coioides* clusters closely with the teleost fish clade (*Takifugu rubripes* and *Oncorhynchus keta*; bootstrap value > 90%), representing strong sequence conservation within this group. Additionally, *E. coioides* clustered as a sister group to salmon fish, confirming their utility as a model organism in teleost comparative endocrinology. The clade of the phylogenetic tree occupied by fishes was positioned adjacent to three clades, such as amphibians and birds, and other mammals. The tree with a separate monophyletic clade of amphibians and birds bridged the evolutionary gap between fish and mammals. The mammalian clade clustered shows high conservation among the species. A distinct subgroup included *Macaca fascicularis* and *Pongo abelii*, which clustered closely, reflecting their primate lineage. Other species of lower eukaryotes and prokaryotes, such as *Escherichia coli*, *Schizosaccharomyces pombe*, *Caenorhabditis elegans*, *Gloeobacter violaceus*, and *Arabidopsis thaliana*, were positioned on more basal branches with low to moderate bootstrap values, highlighting their evolutionary divergence from vertebrate AVT peptide sequences (Figure 3) and, notably, suggesting ancient origins of the precursor peptide with possible non-neuroendocrine roles.

High sequence identity of AVT peptide across different taxa signifies the functional divergence of the protein with unique domain architecture. The proteins IT-1 and VNVT from teleosts and mammals retain neuroendocrine signaling similar to AVT function. The proteins from cyanobacteria (BioD), nitrogen-fixing bacteria (FixB), and *A. thaliana* (GH3.1) share 100% motif identity with the AVT core and are involved in vitamin biosynthesis, energy production, and auxin metabolism. Thus, the AVT motif has a different function, possibly repurposed for regulatory, metabolic, or structural roles. The protein Tnfrsf1b from rats has a 75% motif identity implicated in immune signaling. In rice, the LYP6 protein is involved in the pathogen-defense mechanism. Thus, the analyses reveal the adaptive role of AVT-like domains under strong evolutionary pressure. However, motif conservation does not guarantee functional conservation. The repurposing of the AVT motif across diverse taxa reveals evolutionary flexibility that highlights the importance of domain-level analyses together with sequence identity when predicting function.

### 2.6. Gene Ontology Analyses

**Figure 4:**
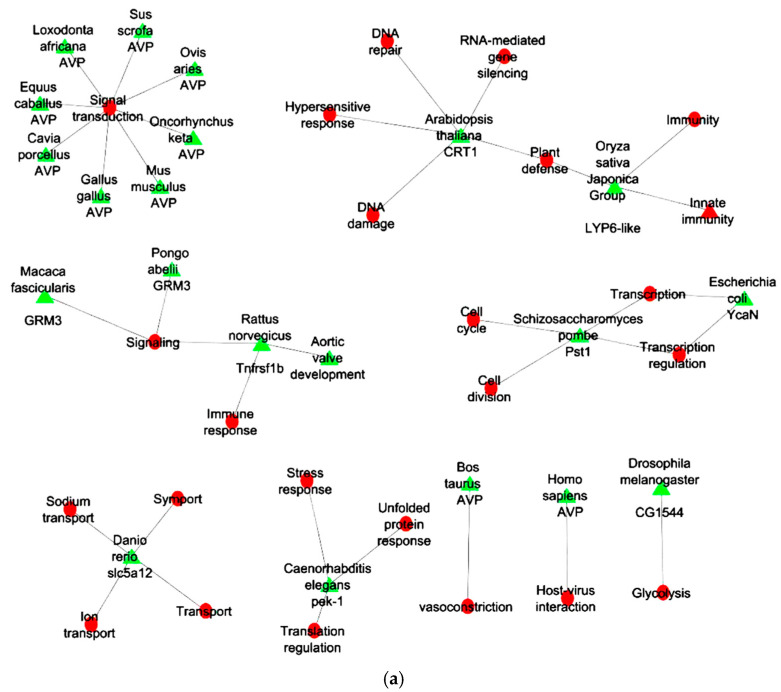

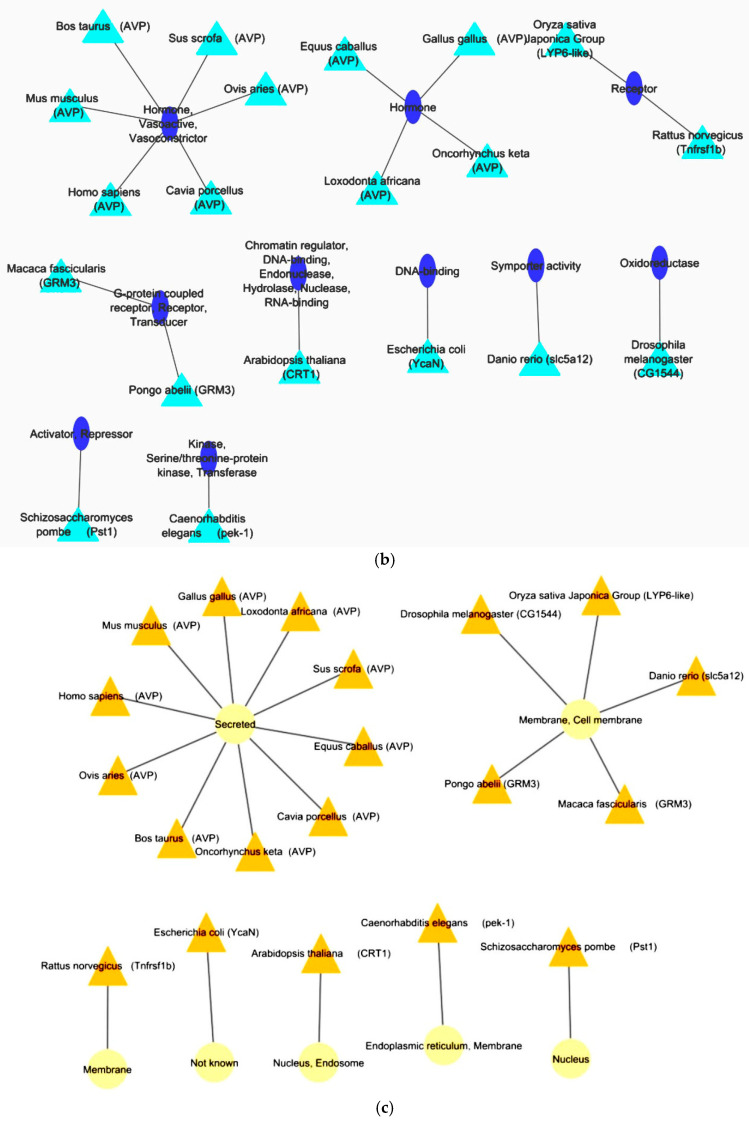
(**a**) Gene ontology analyses representing the biological process. The green circle represents species and the red circle represents their corresponding biological function. (**b**) Interaction network reveals the molecular function of the gene from different taxa. The triangle represents genes from different species involved in different molecular functions (blue color). (**c**) Gene enrichment analyses represents the cellular component. Triangles represent species and their corresponding proteins. Yellow circles represent cellular localization and lines connect each species and its protein to its known cellular localization.

From GO analyses the genes related to AVT peptide sequence from *E. coioides* were functionally enriched. Analyses revealed the genes across different genera were involved in conserved functional role of AVT-related signaling pathways. The AVP from mammals is a direct homolog of AVT, forming a separate cluster involved in signal transduction, vasoconstriction, and host–virus interaction. MORC1 from *A. thaliana* has been involved in several biological process such as “DNA damage”, “DNA repair”, “plant defense”, “hypersensitive response”, “RNA-mediated gene silencing”, “stress response and immune defense”, suggesting analogous roles for AVT-regulated pathways in coordinating responses to external stimuli. In *S. pombe* the PST1 links AVT-like regulatory systems to fundamental process such as processes such as “cell cycle”, “cell division”, “transcription”, and “transcription regulation”. The pek-1 from *C. elegans* involved in “translation regulation”, “stress response”, and “unfolded protein response” supports a role for AVT in modulating cellular homeostasis under environmental challenges. *Tnfrsf1b* from *R. norvegicus* is involved in “immune response”, “aortic valve development”, and “signaling”, representing their role is similar to AVT-related genes. GRM3 is known as a metabotropic glutamate receptor [26] that can specifically interact with G protein and secondary messengers and regulate cellular activity. Thereby, GRM3 from *M. fascicularis* and *P. abeliiis* is involved in “synaptic signaling”, suggesting neuroendocrine involvement, possibly regulated by AVT-like peptides. In *D. rerio*, the slc5a12 gene was involved in “ion transport”, “sodium transport”, and “symport”, which reflects the osmoregulatory processes akin to the function of AVT peptide sequence. The CG1544 gene of *D. melanogaster* involved in glycolysis represents a role in AVT-regulated physiological conditions requiring energy flow. The YcaN gene from *E. coli* was involved in transcription regulation with potential in AVT-like signaling that influences under stress or interaction with hosts (Figure 4a).

The enrichment of the molecular function highlights the biological role of AVT and related genes from different taxa. The AVP gene present in vertebrates has functions such as a “hormone” with “vasoactive” and “vasoconstrictor” properties. The GRM3 gene encodes for G-protein-coupled receptors, suggesting their role in signal transduction. Tnfrsf1b and LYP6-like gene are predicted for receptor activity, indicating that AVT-related genes contribute to cell signaling and immune response mechanisms in both animals and plants. CRT1 and YcaN are involved in DNA-binding, endonuclease, hydrolase, and RNA-binding activities. These molecular functions suggest AVT-linked genes may also participate in gene regulation, chromatin remodeling, and transcriptional control, particularly in plant defense or bacterial stress responses. Pst1 acts as an activator and repressor, emphasizing its role in transcriptional control. The pek-1 gene shows serine/threonine kinase activity during stress and unfolded protein response. The slc5a12 gene contributes to symporter activity, indicating involvement in ion and nutrient transport. The CG1544 gene functions as an oxidoreductase, suggesting a role in metabolic balance and redox signaling (Figure 4b).

The cellular component analyses indicate AVP is the major cluster, which is highly conserved across vertebrates and exhibits secretion in 11 species. In six species, protein (LYP6-like, CG1544, slc5a12, GRM3, and Tnfrsf1b) localization was displayed in the cell membrane. Pek-1 is localized in both the endoplasmic reticulum and the membrane. CRT1 is localized in both the nucleus and endosome. Pek-1 is involved in protein folding or stress responses while CRT1 is suggested to be involved in nucleocytoplasmic transport or signaling. Pst1 protein of yeast shows nuclear localization. YcaN localization remains unpredictable, indicating further experimental validation is needed (Figure 4c).

### 2.7. KEGG Analyses

**Figure 5:**
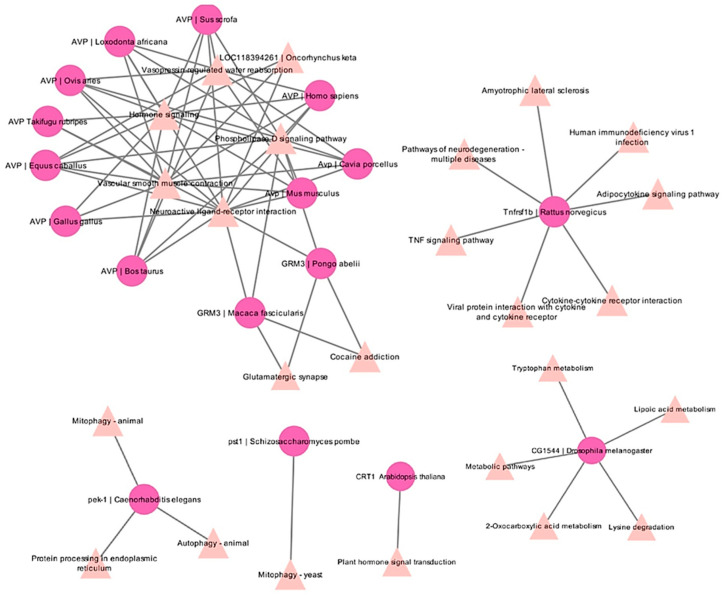
The gene-pathway interaction network illustrates the functional associations between various genes across species and their corresponding KEGG pathways. Nodes in pink circles represent gene-species pairs, while red triangles denote KEGG pathways. Edges indicate functional participation or regulatory involvement of the gene in the respective pathway.

The pathway analyses reveal the functional associations between various genes across species and their corresponding KEGG pathways. The highly conserved gene AVP, across multiple mammalian and vertebrate species, shows extensive interactions with five conserved pathways such as “phospholipase D signaling pathway”, “neuroactive ligand-receptor interaction”, “hormone signaling”, “vascular smooth muscle contraction”, and “vasopressin-regulated water reabsorption”. Tnfrsf1b from *R. norvegicus* is involved in six distinct pathways such as “TNF signaling”, “cytokine–cytokine receptor interaction”, “HIV-1 infection”, and “ALS”. CG1544 from *D. melanogaster* is involved in five metabolic pathways, indicating its role in amino acid degradation and the energy metabolism pathway. MORC1 from *A. thaliana* is involved in the hormone signal transduction pathways that are essential for auxin metabolism and plant development. The gene (pst1 and pek-1) from yeast and *C. elegans* is associated with autophagy and mitophagy, representing their role in cellular stress response and repair mechanisms (Figure 5).

## 3. Discussion

AVT, a neuropeptide from *E. coioides*, has been reported for the synergistic role during steroidogenesis to enhance estradiol biosynthesis for early brain development [9]. Also, studies have supported its role as a hormone network for coordinating reproduction and neural development. However, the functional annotation, particularly the diversity and evolutionary significance of AVT across different taxa, remains elusive. In the present study, the evolutionary, functional, and antimicrobial characteristics of the AVT were investigated. This will provide insight into the highly conserved nature of AVT and its homologs in diverse biological systems.

The identification of the AMP motif within the conserved sequence of AVT and its homologous sequences revealed the GIRQCMSCGPGDRGR segment. The peptide sequence exhibited a dual role as an antimicrobial peptide beyond its endocrine function. The physicochemical analyses revealed that the cationic peptide (+2 charge) has a hydrophilic and amphipathic nature favorable for interactions with bacterial membranes. AMPs, on interaction with the electronegative cell membranes, lead to pore formation. The cationic peptide penetrates inside the cell, resulting in cell lysis or disruption [27,28]. Additionally, the cysteine residues in the peptide sequence enable structural stability through disulfide bond formation. AVT aligns structurally with defensins (RC-101 and Retrocyclin-1) in its disulfide-bridge-forming cysteine residues, its cationic surface, and short length, all of which are hallmark features of antimicrobial peptides. In vitro investigations have shown that RC-101 and Retrocyclin-1 have broad-spectrum antimicrobial activities as such as antibacterial, antiviral, and antibiofilm. Additionally, the peptides also have antitoxin properties. The peptides investigated with human epithelia resulted in high safety and tolerability in host cells. Although AVT has not yet been validated through in vitro studies, by its strong structural and sequence-based similarity to AMPs it offers a compelling rationale for its predicted antimicrobial activity.

In a study with nodule-specific cysteine-rich (NCR) peptides, the replacement of cysteine (C) with serine (S) removes the ability to form disulfide bonds. This modification in legume AMP influences their activity against *S. meliloti* [29]. Therefore, it is envisaged that the AMP sequence with positively charged arginine residues enhances the electrostatic interactions with Gram-negative microbial membranes. Hexapeptides rich in arginine (R) and tryptophan (W), both in linear and cyclic forms, have been widely studied and considered as promising candidates as antimicrobials [30]. The investigation supports the present study, indicating that the segment of AVT is predicted as a cell-penetrating peptide (CPP). The predicted AMP can mediate membrane permeabilization, with the potential to internalize into cells without leakage. From the MEME analyses, a conserved 11-aa motif (RQCMSCGPGDR) was revealed with charged and structurally flexible residues acting as a binding interface or signal modulator. The motif is predicted to contribute to antimicrobial, osmoregulatory, or protein-interacting roles of AVT, especially under stress or pathogen invasion. Thus, the presence of AVT conservation across different taxa may serve as a candidate for synthetic peptide design targeting microbial infections or inflammatory pathways. These findings are envisaged as hypothesis generating rather than conclusive. Future studies involving experimental validation are essential to confirm any antimicrobial or antiviral activity

The sequence similarity revealed AVT peptide is conserved across distant taxonomical groups from teleost fish to mammals and their lower organisms, such as plants and bacteria. The AVT peptide sequence shared 100% identity with White sucker, Thale cress, and Cyanobacterium, which suggests a conserved motif evolved due to the strong selective pressure essential for their function. Moderate 64–85% conservation of the peptide in mammals and other vertebrates represents species-specific divergence of the AVT sequence. This highlights that the AVT peptide has a core function essential for osmoregulation, neuroendocrine signaling, and social behavior in other species. However, ML phylogenetic analyses reveals evolutionary descent, lateral gene transfer, profiles, and patterns for the presence and absence [31] of the AVT genes across different taxa. The circular tree showed the *E. coioides* clustered with salmonids and pufferfish, indicating functional conservation of AVT peptide with marine and freshwater teleosts. The evolutionary placement of *E. coioides* in this group reinforces its importance as a suitable model for understanding the role of AVT in reproductive and stress-related pathways in aquaculture species.

The branch was subsequently grouped with other taxa of amphibians, birds, and mammals. The hierarchical arrangement of the branch reveals that the mammalian AVP gene originated from a common ancestral gene. Previous studies have shown that AVT and AVP are members of the AVP/oxytocin (OT) superfamily of peptides responsible for the regulation of social behavior, cognition, and emotion [1]. Comparative studies have revealed the social behavior neural network (SBNN) is distributed with AVT/AVP peptides and receptors that control different social behavior properties [32]. Thus, the analyses of AVT peptide from *E. coioides* further supports their conserved role and evolutionary importance in neuroendocrine signaling, stress response, and social behavior across different taxa. The presence of the basal branches with lower bootstrap values indicates bacteria and plant peptides as the ancient variants or precursors that evolved before the divergence of eukaryotes. Thus, the role of AVT-like peptides in prokaryotes and plants could be linked to neo-functionalization such as cell signaling, antimicrobial activity, or stress adaptation, rather than neuroendocrine functions.

The study highlights the structural, sequence conservation of AVT across diverse taxa, and does not support complete functional conservation. Previous studies have reported that AVP and OXT evolved from a single vasopressin-like peptide from invertebrates. The physiological role of these proteins has diverged throughout vertebrate evolution. AVT from the teleost group has been reported for its established roles in reproduction, including steroidogenesis and courtship behavior [33]. The functional roles of AVT/AVP family members in teleosts show variation across the species, suggesting partial divergence or species-specific adaptation depending on ecological and hormonal factors. These observations emphasized a restrained interpretation of functional annotations across distantly related species such as plants and bacteria, where similar sequences can have paralogous protein functions.

The pleiotropic functions of the AVT and the homologous genes from other taxa are revealed through GO and KEGG pathway analyses. In vertebrates, AVP and AVT are well known for vasoconstriction, osmoregulation, and neuroendocrine signaling [34]. From the KEGG network, it was observed that the AVP-centered cluster reveals strong functional conservation of hormonal signaling pathways in vertebrates. The genes CRT1 and Pst1 from plants and yeast exhibit functional analogy to AVT, such as stress response, transcriptional regulation, and immune defense. The genes like CRT1 and CG1544 are involved in critical species-dependent biological processes, indicating specialized adaptations during evolution. The identified role of GRM3 and Tnfrsf1b in synaptic and immune signaling further emphasizes the functional role of AVT/AVP pathways in higher organisms. The KEGG cluster shows that Tnfrsf1b from *R. norvegicus* is involved in diverse signaling and disease pathways. Hence, Tnfrsf1b is determined to be a potential therapeutic target to treat inflammatory and neurodegenerative diseases. The results very well corroborate with the recent reports on ovarian cancer (OC) and androgenetic alopecia (AGA) that Tnfrsf1b is a novel therapeutic target [35,36]. In this study, through GO and KEGG pathway analyses, the evolutionary presence of AMP-related motifs and their possible functional divergence were traced across different taxa. While GO and KEGG annotations provide essential insights into related pathways, the functional annotation transfer between distantly related species has limitations. Therefore, the functional predictions using GO and KEGG pathway analyses presented here are interpreted as evolutionary indicators, which require further evaluation.

Overall, this study sheds light on the multifunctionality and evolutionary stability of the AVT peptide. The findings not only affirm AVT’s potential role in vertebrate neuroendocrine systems but also propose a neo-functionalization role for AVT peptide in antimicrobial, stress response, or host–pathogen interaction functions across diverse life forms. These hypotheses require future experimental validation to assess the antimicrobial activity and in vivo functional relevance.

## 4. Materials and Methods

### 4.1. Sequence Retrieval

The target protein for the study, arginine vasotocin (AVT) from *E. coioides,* was retrieved from the NCBI GenBank database.

### 4.2. Prediction and Analyses of Antimicrobial Peptide

The AVT sequence from *E. coioides* was used for the prediction of an antimicrobial peptide. The Antibp server (https://webs.iiitd.edu.in/raghava/antibp/index.html, accessed on 2 June 2025) was used for the prediction of the antibacterial peptides in a protein sequence. The prediction was performed by using QM, ANN, and SVM-based methods using binary patterns of peptide sequences. The overall accuracy of this server is ~92.11%. The predicted peptides are significant for designing peptide-based antibiotics [37]. The peptide sequence was determined for various physical and chemical parameters using the ProtParam tool (https://web.expasy.org/protparam/, accessed on 2 June 2025) available from ExPASy [38]. Several parameters of the peptide, such as their molecular weight, pI, composition of the amino acids, aliphatic index, and grand average of hydropathicity (GRAVY), were computed. The predicted peptide was searched for the similarity in APD3 available from https://aps.unmc.edu/AP/, accessed on 2 June 2025 [39].

### 4.3. Domain Identification and Functional Annotation

Conserved domains within the AVT sequence were identified using the MEME suite (Multiple Em for Motif Elicitation) Version 5.5.8. Annotation helped to confirm the neurohypophysial hormone domain, signal peptide features, and uncharacterized sequences. MEME represents the position-dependent and letter-probability matrices of the motifs. The probability of each amino acid encoding for the letter present in the particular position is represented through the pattern. MEME HTML output displays the e-value, which represents the smaller the e-value with the more likely the pattern is the top hit [40].

### 4.4. Multiple Sequence Alignment (MSA)

The peptide sequence predicted from AVT protein sequence was used as the search query to identify the homologous sequence and determine evolutionary conservation. The query was given as input in the BLASTp (https://blast.ncbi.nlm.nih.gov/Blast.cgi, accessed on 14 August 2025) program available with the NCBI server. Using the BLAST tool (version BLAST+ 2.17.0), the query sequence was searched against the Uniprot protein database and all other parameters were set to default.

The retrieved sequences that are homologous to AVT sequences were submitted to the Clustal Omega (2.3.0+a8aa7244) (https://www.ebi.ac.uk/Tools/msa/clustalo/, accessed on 2 June 2025) web server [41]. MSA was performed using default settings and the output file was manually curated. The default parameters, such as a gap opening penalty of 10 and gap extension penalty of 0.2, was used for the alignment process. For manual curation of the sequence, the poorly aligned or incomplete sequences were excluded using a threshold of >33% sequence coverage and >50% identity for inclusion. For MSA, the complete FASTA sequence of all the proteins were downloaded. The sequences were imported to the Clustal X tool and the alignment was performed, the conserved residues and domain stretches among the sequences, particularly within the AVT peptide and in other homologous sequences, were manually curated [42].

### 4.5. Phylogenetic Analysis

To construct the phylogenetic tree for AVT and homologs, MEGA11 software was employed [43]. The 36 sequences for analyses were imported into the MEGA tool workspace. The alignment obtained was used further for constructing the AVT phylogenetic tree. Maximum likelihood method was performed of the alignment using the Select Jones–Taylor–Thornton (JTT) substitution model with 1000 bootstrap replicates used to assess the reliability of the tree branches [44]. For tree inference, the nearest neighbor interchange ML heuristic method was selected. The tree file constructed was imported into the online tool available from https://itol.embl.de (accessed on 2 June 2025) (Version 7.2). The online tool Interactive Tree Of Life (iTOL) helps to visualize, edit, and annotate the phylogenetic trees [45].

### 4.6. Gene Ontology (GO) Analysis

To understand the functional classification of AVT and their homologous genes, GO analyses were performed using an online annotation tool, DAVID [46]. The tool predicts the functional GO annotation of the genes from different taxa related to AVT. The three categories of GO, such as biological processes, molecular function, and cellular component, were annotated using DAVID. The functionally enriched genes resulting from the three categories [47] were constructed into an interaction network. All default parameters were used for analysis. The network was visualized using Cytoscape (version 3.10.1). The genes from different taxa and their involvement in the pathway were enriched using the KEGG database [48] and represented using an interaction network.

## 5. Conclusions

This study highlights the evolutionary conservation of AVT peptide across different taxa. The AVT peptide sequence is highly conserved among different taxa of life and describes the peptide as multifunctional and essential for various functions. The tree of life analyses states that the AVT motif is conserved across vertebrates, fishes, amphibians, plants, and bacteria, suggesting that the peptide has been maintained under strong evolutionary pressure to maintain critical physiological and biological functions. Although the AVT motif is evolutionarily conserved across kingdoms, the proteins harboring this motif in plants and prokaryotes are functionally unrelated to neuroendocrine peptides, suggesting a potential neo-functionalization of this domain to support alternative physiological roles such as stress response or host–pathogen interaction. Functional annotation through GO and KEGG analyses supports that AVT is involved in stress response, immune signaling, and receptor-mediated pathways. Despite their well-established role in neuroendocrine signaling, osmoregulation, and social behavior, AVT-like peptides also possess a conserved motif that has been predicted to exhibit characteristic features as an antimicrobial peptide. These findings not only expand our understanding of the biological significance of AVT but also open avenues for exploring its use as a synthetic peptide design for targeting microbial infections and inflammation. Future in vitro and in vivo validation will be essential to confirm the antimicrobial activity and regulatory potential of AVT-derived peptides in physiological and pathological contexts.

## Figures and Tables

**Table 1:** Physicochemical properties of the peptide sequence obtained from ProtParam and HeliQuest server.

Property	Value	Composition
Molecular Weight	1592.83 Da	
Theoretical Isoelectric Point (pI)	9.02	
Net Charge (z)	2	
Positively Charged Residues	3	Arg(3)
Negatively Charged Residues	1	Asp(1)
Mean Hydrophobicity (⟨H⟩)	0.185	Mildly hydrophobic
Hydrophobic Moment (⟨µH⟩)	0.21	Low amphipathicity
Polar Residues + Glycine	10 (66.67%)	GLN(1), SER(1), GLY(4), Arg(3), Asp(1)
Nonpolar Residues	5 (33.33%)	LEU(1), VAL(1), ALA(1), ILE(1), MET(1)

**Table 2:** AMP-like peptide sequence from AVT of *E. coioides* showing sequence similarity with different genera.

Protein Name	Scientific Name	Common Name	Percent Identity	Accession ID
Isotocin-neurophysin (IT-1)	*Catostomus commersonii*	White sucker	100.00%	P15210
Indole-3-acetic acid-amido synthetase (GH3.1)	*Arabidopsis thaliana*	Thale cress	100.00%	O82333
Dethiobiotin synthetase (BioD)	*Gloeobacter violaceus*	Cyanobacterium	100.00%	Q7NFL5
Collagen alpha-1 (Col4A1)	*Drosophila melanogaster*	Fruit fly	100.00%	P08120.3
NADH-quinone oxidoreductase subunit C/D	*Psychrobacter arcticus*	Arctic bacterium	100.00%	Q4FU62
NADH-quinone oxidoreductase subunit C/D	*P. cryohalolentis*	Cold-tolerant bacterium	100.00%	Q1QD95
Vasotocin-neurophysin VT 1	*Takifugu rubripes*	Japanese pufferfish	93.33%	O42499
IT 1	*Oncorhynchus masou*	Masu salmon	92.31%	Q07663
LysM domain-containing GPI-anchored (LYP6)	*Oryza sativa Japonica*	Rice	88.89%	Q69T51
Sodium-coupled monocarboxylate transporter 2 (slc5a12)	*Danio rerio*	Zebrafish	87.50%	Q7T384
Protein MICRORCHIDIA 1 (MORC1)	*Arabidopsis thaliana*	Thale cress	85.71%	Q84WV6
Metabotropic glutamate receptor 3 (GRM3)	*Macaca fascicularis*	Crab-eating macaque	85.71%	Q1ZZH1
GRM3	*Pongo abelii*	Sumatran orangutan	85.71%	Q5RAL3
Paired amphipathic helix protein (PST1)	*Schizosaccharomyces pombe*	Fission yeast	85.71%	Q09750
HTH-type transcriptional regulator (YcaN)	*Escherichia coli K-12*	E. coli	85.71%	P75836
Vasotocin-neurophysin VT 2	*Oncorhynchus keta*	Chum salmon	80.00%	P16042
Neurophysin 2	*Struthio camelus*	Ostrich	78.57%	P21916
Arginine decarboxylase (ArgD)	*Bacillus cereus*	Bacillus cereus	77.78%	Q819L4
ArgD	*Bacillus anthracis*	Anthrax bacterium	77.78%	Q81MS2
TNF receptor superfamily member 1B (Tnfrsf1b)	*Rattus norvegicus*	Rat	75.00%	Q80WY6
Uncharacterized protein 5	*Haliotis asinina*	Sea snail	75.00%	P86728
Protein FixB	*Azotobacter vinelandii*	Soil bacterium	75.00%	P53574
Translation initiation factor 2 kinase (pek1)	*Caenorhabditis elegans*	Nematode	72.73%	Q19192
Vasotocin-neurophysin VT (VNVT)	*Pelophylax lessonae*	Pool frog	69.23%	P11858
VNVT	*Gallus gallus*	Chicken	66.67%	P24787
Neurophysin 2	*Anser anser anser*	Domestic goose	66.67%	P19630
VNVT	*Bufo japonicus*	Japanese toad	64.29%	P08163
Vasopressin-neurophysin 2-copeptin (VN2C)	*Sus scrofa*	Pig	64.29%	P01183
VN2C	*Bos taurus*	Cow	64.29%	P01180
VN2C	*Homo sapiens*	Human	64.29%	P01185
VN2C	*Mus musculus*	Mouse	64.29%	P35455
VN2C	*Ovis aries*	Sheep	64.29%	P01181
Neurophysin 2	*Loxodonta africana*	African elephant	64.29%	P81768
VN2C	*Cavia porcellus*	Guinea pig	64.29%	P10769
Neurophysin 2	*Equus caballus*	Horse	64.29%	P01182
VN2C	*Balaenoptera physalus*	Fin whale	64.29%	P01184

## Data Availability

Data are contained within this article. Raw data are available on request from the corresponding author.

## Abbreviations

The following abbreviations are used in this manuscript:

AVT	Arginine vasotocin
AVP	Arginine vasopressin
IT-1	Isotocin-neurophysin
GH3.1	Indole-3-acetic acid-amido synthetase
BioD	Dethiobiotin synthetase
LYP6	LysM domain-containing GPI-anchored
MORC1	Protein MICRORCHIDIA 1
PST1	Paired amphipathic helix protein
YcaN	HTH-type transcriptional regulator
ArgD	Arginine decarboxylase
VNVT	Vasotocin-neurophysin VT
VN2C	Vasopressin-neurophysin 2-copeptin
*Tnfrsf1b*	TNF receptor superfamily member 1B
GRM3	Metabotropic glutamate receptor 3
OC	Ovarian cancer
AGA	Androgenetic alopecia
slc5a12	Sodium-coupled monocarboxylate transporter 2
SBNN	Social behavior neural network
OT	AVP/oxytocin
AMP	Antimicrobial peptide
GO	*Gene ontology*
iTOL	Tree of life
NJ	Neighbor-joining
MEME	Multiple Em for Motif Elicitation
